# Metastasis of Breast Carcinoma to the External Auditory Canal: Report of an Unusual Case and Literature Review

**DOI:** 10.5334/jbr-btr.853

**Published:** 2015-09-15

**Authors:** S. Sari, B. Battal, V. Akgun, M. Salih Deveci

**Affiliations:** 1 Department of Radiology, Gulhane Military Medical School, Etlik, Ankara, Turkey; 2 Department of Pathology, Gulhane Military Medical School, Etlik, Ankara, Turkey

**Keywords:** Breast neoplasms, metastases

## Abstract

We present a case of metastasis of the external auditory canal (EAC) from a primary breast carcinoma in a 53-year-old female with a review of the literature. The patient had been diagnosed with a primary carcinoma 4 years previously. The metastasis had developed recently in her left EAC and presented as a bulky, fleshy, bleeding mass. The mass was causing hearing loss on the left due to complete obstruction in the left EAC. The mass was incompletely removed with a surgical operation and histopathologically metastasis was proven. Although there are few case reports in the literature of various cancers metastasizing to the EAC, metastasis in the EAC from the breast carcinoma is exceedingly rare and only one case has been reported in the literature so far.

Metastases to the external auditory canal (EAC) are exceptionally rare. These metastases almost always occur in the advance stages of the disease process, but rarely EAC mass may be the first manifestation of the metastatic carcinoma [1]. Only 11 cases of metastatic tumors in the EAC have been reported in the literature [1,2,3,4,5,6,7,8,9,10,11]. However, radiologic imaging findings including high resolution computed tomography (HRCT), magnetic resonance imaging (MRI) and diffusion-weighted imaging (DWI) were not presented comprehensively, and radiological differential diagnoses of the metastasis from primary neoplastic tumors and other benign conditions of the EAC were not discussed. In this report, we aimed to present radiologic imaging findings of the left EAC metastasis from breast carcinoma, discuss differential diagnoses based on the imaging features and review of the pertinent literature.

## Case report

**Figure 1:**
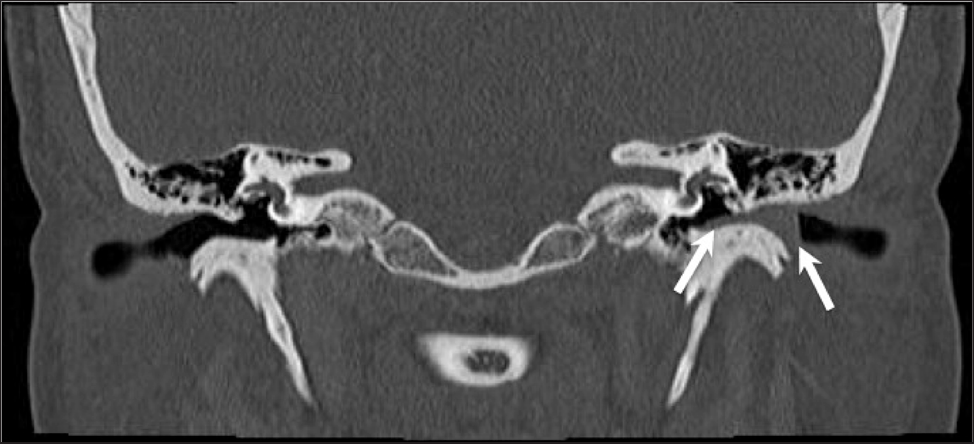
Coronal HRCT image shows totally obstructed left EAC by soft tissue density mass (arrows) without bony EAC erosion or destruction. The middle and internal ear structures are normal.

**Figure 2:**
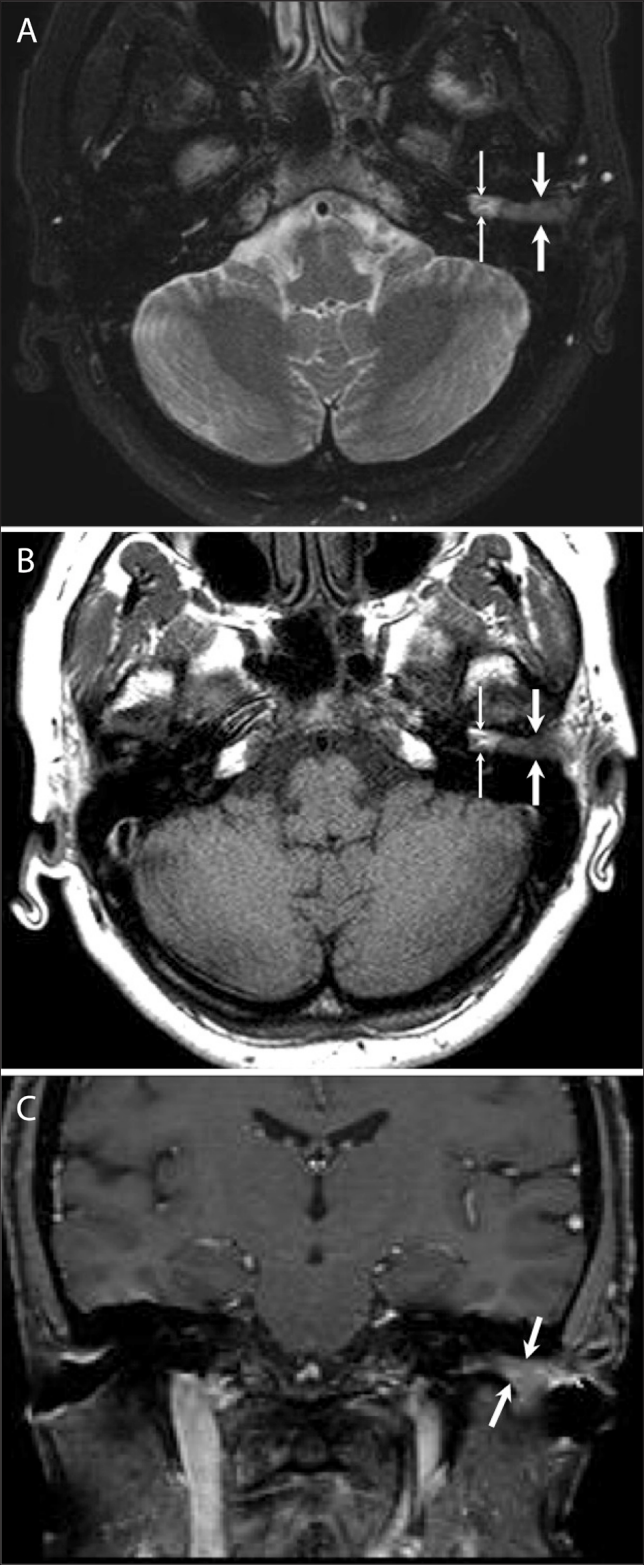
Axial fat-suppressed T2-weighted (A) and T1-weighted (B) images show left EAC soft tissue mass (thick arrows) that is iso-intense with muscle on T1-weighted image, slightly hyperintense on T2-weighted image. There is a small, T1- and T2 hyperintense, hemorrhagic fluid collection (thin arrows) between the mass and the tympanic membrane. Post-contrast coronal fat-suppressed T1-weighted (C) images show intense contrast enhancement in the mass.

A 53-year-old woman with invasive ductal breast carcinoma diagnosed 4 years previously was admitted to our hospital with a history of swelling in her left EAC for 2 weeks and a gradually worsening hearing loss. The patient had undergone right radical mastectomy and followed chemoradiotherapy following the diagnosis. The primary tumor was multifocal (5 different focuses) and located in upper and lower outer quadrants of the breast. The size of the largest tumor focus was 2.2 cm. The immunohistochemical study showed positive Estrogen Receptor. All of the 33 dissected axillary lymph nodes were free of metastasis. Post surgical staging was stage IIB. In follow up, bone and lung metastases appeared and relevant treatment was performed. Initially, she had noticed a moderate hearing loss in her left ear, but mentioned no otalgia, otorrhea, tinnitus, or vertigo. Physical examination revealed complete obstruction of the left EAC by a soft tissue mass. No neurologic symptoms were observed. HRCT of the temporal bone showed that totally obstructed left EAC by soft tissue density mass, but no bony erosion or destruction (Fig. 1). MRI showed well defined, fusiform, soft tissue mass entirely filling the left EAC. The mass was iso-intense with muscle on T1-weighted images, slightly hyperintense on T2-weighted images. Following the administration of the gadolinium based contrast agent homogeneous enhancement was seen. Although the mass was located in both bony and cartilaginous EAC, there was no sign of bony or cartilaginous invasion (Fig. 2). DWI showed slightly hyperintense signal on isotropic trace image and the apparent diffusion coefficient (ADC) map revealed slightly restricted diffusion in the mass. There was a small, non-enhancing hemorrhagic fluid collection, which is hyperintense on both T1- and T2-weighted images, between the mass and the tympanic membrane. The middle and internal ear structures were normal on both MRI and temporal CT images. We reported that the mass may be consistent with primary tumor of the EAC, but due to the history of metastatic breast carcinoma, mentioned EAC metastasis in the differential diagnoses.

**Figure 3:**
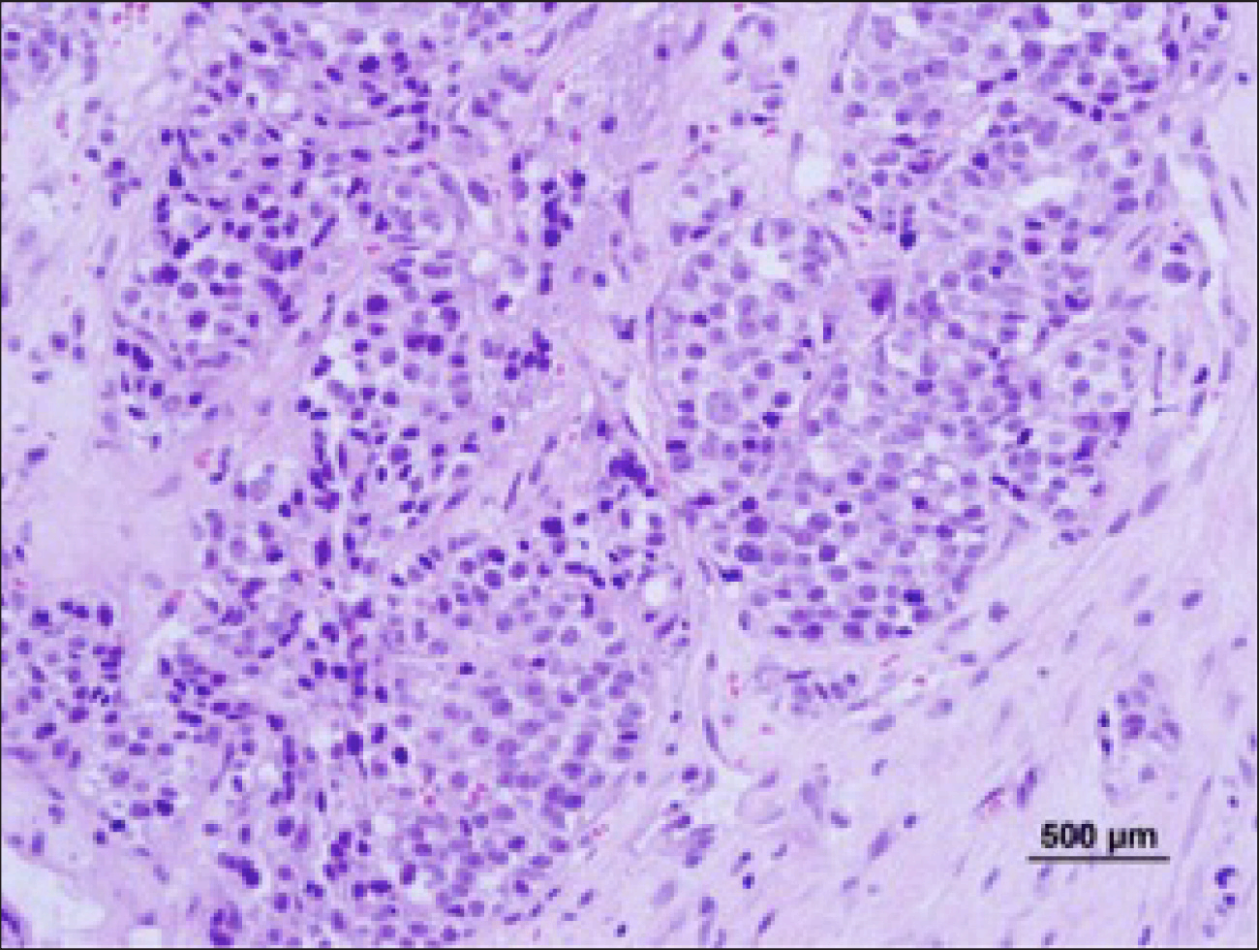
Histological appearance of the resected EAC mass showing high grade ductal carcinoma (Hematoxylin-Eosin, x400).

**Figure 4:**
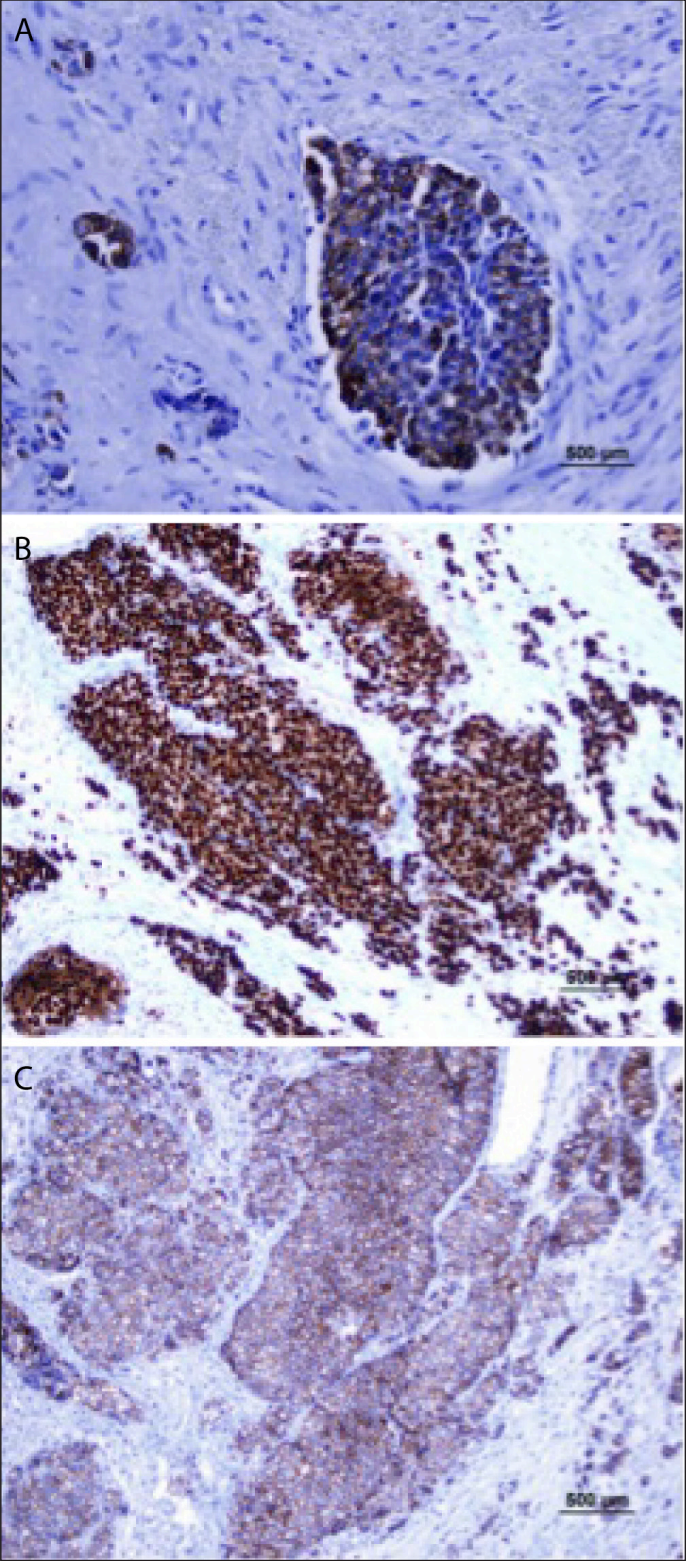
Result of immunohistochemical staining for the EAC mass. A. Positive immunostaining for cytokeratin 7 (x200). B. Positive immunostaining for Estrogen Receptor (x200). C. Positive immunostaining for E-cadherin (x200).

The patient underwent surgical operation by the ENT surgeon and the mass was subtotally removed. Histopathologic examination of specimens revealed metastatic carcinoma that has same histopathologic features with the primary breast carcinoma. Histopathological finding of the excised lesion showed microscopically a infiltrative carcinoma compatible with breast origin (Fig. 3) based on immunohistochemical study result that was positive for Estrogen Receptor (ER), Cytokeratin 7 and E-cadherin (Fig. 4) but negative for C-Erb-b2 and Progesterone receptor (PR). Tumor was composed of atypical cells with large nuclei and prominent nucleoli in solid pattern. There was no ductal or tubular structure. Positivity of both ER and E-Cadherin also supported ductal type when compared to lobular carcinoma of the breast.

## Discussion

EAC can be affected various lesions including congenital, inflammatory and benign or malignant tumors. The most common congenital lesion is EAC atresia. Inflammatory lesions such as malignant otitis externa and osteomyelitis can affect EAC. Exostosis, osteoma, and adenoma are the most common benign lesions of the EAC. Carcinomas of EAC are very rare. Squamous cell carcinoma (SCC) is the most common malignant tumor affecting the EAC. Basal cell carcinoma, adenoid cystic carcinoma, adenocarcinoma, melanoma, various sarcomas and metastases are among the other malignancies within the EAC. Miscellaneous conditions such as accumulated earwax and cholesteatoma can also affect the EAC [1,12,13,14,15].

The malignant and benign lesions in the EAC may have similar clinical features, therefore, the clinical differentiation of those lesions may be difficult. The clinician and the radiologist should consider various benign and malignant lesions in the differential diagnoses of a mass in the EAC. CT is the primary tool for diagnostic evaluation and in the differentiation of the exostoses and osteomas. Osteomas of the EAC are discrete, pedunculated bone lesions arising along the tympano-squamous suture while exostoses of external auditory canal are broad based elevations of bone usually multiple and bilaterally symmetric, involving the tympanic bone [13,14,16,17]. The aural polyp, adenoma, foreign body granuloma, schwannoma, melanoacanthoma, hemangioma and hemangiopericytoma are other benign lesions of the EAC [1,12,13,14]. Although, some benign lesions may show similar radiologic appearance with malignant ones, these soft tissue tumors can be more precisely evaluated with magnetic resonance imaging including contrast enhanced sequences and DWI.

Cholesteatoma is an inflammatory lesion of the temporal bone that uncommonly involves the EAC [18]. EAC cholesteatoma must be differentiated from malignant tumors and malignant otitis externa. EAC cholesteatoma is a slowly progressing chronic disease with a soft tissue density eroding any wall of the EAC. On the other hand, malignant otitis externa is a rapidly progressive disease and a diffuse process involving most of the EAC [18,19]. Temporal bone CT shows EAC cholesteatoma as a soft-tissue mass within the EAC, with adjacent bone erosion. Bone fragments may be present within the mass. The cholesteatoma may extend into the mastoid or middle ear, or it may involve the facial nerve canal or tegmen tympani. In MRI, malignant otitis externa shows soft tissue enhancement on contrast-enhanced studies, whereas there is no enhancement in cholesteatomas. EAC cholesteatoma show prompt diffusion restriction, whereas malignant otitis externa does not show restriction in DWI [20]. But, some malignant lesions that have high cellularity and high nucleus/cytoplasm ratio may show diffusion restriction in DWI. In this situation, contrast enhancement of the tumor is an important feature for its differentiation from the EAC cholesteatoma.

Although, most of the malignant tumors arising from the EAC are primary tumors such as squamous cell or basal cell carcinoma, metastatic tumor should also be included in the differential diagnoses of a soft tissue mass in the EAC. Metastatic disease in the EAC is exceedingly rare, and only 11 cases were described in the literature. The primary tumors were located in the kidneys in 3 cases, in the esophagus in 2 cases, and in the lung, breast, liver, colon, rectum, and prostate [1,11].

In our case, a metastatic breast carcinoma was presented as a bulky, fleshy, bleeding EAC mass. HRCT of the temporal bone showed totally obstructed left EAC by soft tissue density mass, but no bony erosion or destruction. Due to these CT findings and homogeneous contrast enhancement of the mass on MRI we did not consider the EAC choleastatoma. However, contrast enhancement and restricted diffusion could not help the differentiation of the primary or metastatic malignant neoplasm. MRI and HRCT precisely showed the extent of the tumor and the normal condition of the surrounding structures and other ear compartments. The definitive diagnosis could be made after excisional biopsy. Most of the EAC lesions can be diagnosed clinically, however imaging is often required to evaluate the extent of the lesion, feasibility for surgery, differential diagnosis and to rule out complications.

In conclusion, although HRCT is a good tool to assess EAC abnormalities especially located in bony part and bony lesions, MRI is more useful in differential diagnosis and the evaluation the extension of the soft tissue lesions affecting the EAC. DWI can provide some additional information for the differential diagnosis in lesions such as cholestatoma and benign and malignant soft tissue neoplasms affecting the EAC. Although metastasis to EAC is exceedingly rare, clinicians and radiologist should be kept in the mind the metastasis in the differential diagnoses of the EAC mass.

## Competing Interests

The authors declare that they have no competing interests.

## References

[1] Vasileiadis I, Kapetanakis S, Vasileiadis D, Petousis A, Karatzas T (2013). External auditory canal mass as the first manifestation of a bronchogenic carcinoma: report of a rare case. Ann Otol Rhinol Laryngol.

[2] Michaelson PG, Lowry TR (2005). Metastatic renal cell carcinoma presenting in the external auditory canal. Otolaryngol Head Neck Sur.

[3] Cumberworth VL, Friedmann I, Glover GW (1994). Late metastasis of breast carcinoma to the external auditory canal. J Laryngol Otol.

[4] Yasumatsu R, Okura K, Sakiyama Y, Nakamuta M, Matsumura T, Uehara S, Yamamoto T, Komune S (2007). Metastatic hepatocellular carcinoma of the external auditory canal. World J Gastroenterol.

[5] Goldman NC, Hutchison RE, Goldman MS (1992). Metastatic renal cell carcinoma of the external auditory canal. Otolaryngol Head Neck Surg.

[6] Ingelaere PP, Simpson RH, Garth RJ (1997). Metastatic renal cell carcinoma presenting as an aural polyp. J Laryngol Otol.

[7] Carson HJ, Krivit JS, Eilers SG (2005). Metastasis of colonic adenocarcinoma to the external ear canal: an unusual case with a complex-pattern of disease progression. Ear Nose Throat J.

[8] Carr S, Anderson C (2009). Metastatic rectal adenocarcinoma in the external auditory canal. J Laryngol Otol.

[9] Shrivastava V, Christensen R, Poggi MM (2007). Prostate cancer metastatic to the external auditory canals. Clin Genitourin Cancer.

[10] Imauchi Y, Kaga K, Nibu K, Sakuma N, Iino Y, Kodera K (2001). Metastasis of cervical esophageal carcinoma to the temporal bone—a study of the temporal bone histology. Auris Nasus Larynx.

[11] Lollar KW, Parker CA, Liess BD, Wieberg J (2010). Metastatic esophageal adenocarcinoma presenting as an external auditory canal mass. Otolaryngol Head Neck Surg.

[12] Devaney KO, Boschman CR, Willard SC, Ferlito A, Rinaldo A (2005). Tumours of the external ear and temporal bone. Lancet Oncol.

[13] Vogl TJ, Balzer J, Mack M, Steger W (1998). Differential Diagnosis in Head and Neck Imaging, a Systemic Approach to the Radiologic Evaluation of the Head and Neck Region and the Interpretation of Difficult Cases.

[14] Chatra PS (2011). Lesions in the external auditory canal. Indian J Radiol Imaging.

[15] Lee BJ, Bae SC, Lee JH, Park KH (2012). A case of Basal cell carcinoma of external auditory canal. Korean J Audiol.

[16] Fenton JE, Turner J, Fagan PA (1996). A histopathologic review of temporal bone exostoses and osteoma. Laryngoscope.

[17] Agarwal A, Deschler DG, Baker KB (1999). Exostoses of the external auditory canal. Am J Otol.

[18] Heilbrun ME, Salzman KL, Glastonbury CM, Harnsberger HR, Kennedy RJ, Shelton C (2003). External auditory canal cholesteatoma: clinical and imaging spectrum. AJNR Am J Neuroradiol.

[19] Applebaum EL, Duff BE, Fu YS, Wenig BM, Abemayor E, Wenig BL (2001). Ear and temporal bone, I: Clinical considerations for non-neoplastic lesions of the ear and temporal bone. Head and Neck Pathology with Clinical Correlations.

[20] Kavanagh EC, Fenton DM, Griesdale D, Graeb DA (2005). MRI of Acquired Cholesteatoma Presenting as a Temporal Lobe Mass. AJR Am J Roentgenol.

